# Efficacy and safety of repetitive transcranial magnetic stimulation in anorexia and bulimia nervosa: a systematic review and meta-analysis

**DOI:** 10.1186/s12991-026-00673-2

**Published:** 2026-06-01

**Authors:** Dalia Kamal Ewis, Haneen Sabet, Yousef Hawas, Wajeeh Hassan, Moaz Elsayed Abouelmagd, Arwa Jader, Dina Essam Abo-elnour, Abdallah Abbas, Mohamed E. G. Elsayed, Carlos Schönfeldt-Lecuona, Ahmed M. Raslan

**Affiliations:** 1 Faculty of Medicine, Beni Suef University, Beni Suef, Egypt; 2 Faculty of Medicine, South Valley University, Qena, Egypt; 3 Faculty of Medicine, Tanta University, Gharbeya, Egypt; 4 Allama Iqbal Medical College, Jinnah Hospital, Lahore, Pakistan; 5 Faculty of Medicine, Cairo University, Cairo, Egypt; 6 Neurosurgery Department, Kufa University, Kufa, Iraq; 7 Faculty of Medicine, Zagazig University, Zagazig, Egypt; 8 Faculty of Medicine, Al-Azhar University, Damietta, Egypt; 9 Department of Psychiatry and Psychotherapy III, University of Ulm, Ulm, Germany; 10 Department of Psychiatry, School of Medicine and Health Sciences, Carl Von Ossietzky University Oldenburg, Oldenburg, Germany; 11 CuraMed Day-Clinic for Psychiatry, Psychosomatic Medicine and Psychotherapy, Neu-Ulm, Germany; 12 Department of Neurological Surgery, Oregon Health & Science University, Portland, OR USA

**Keywords:** Repetitive transcranial magnetic stimulation, rTMS, Transcranial magnetic stimulation, TMS, Eating disorders, Anorexia nervosa, Bulimia nervosa

## Abstract

**Objective:**

This systematic review and meta-analysis aimed to evaluate the efficacy and safety of repetitive transcranial magnetic stimulation (rTMS) in patients with anorexia nervosa (AN) and bulimia nervosa (BN), focusing on its impact on psychological, psychopathological, neurocognitive, and behavioral outcomes.

**Methods:**

A comprehensive literature search was conducted in PubMed, Scopus, Web of Science, and Cochrane CENTRAL from inception to May 2025. Eligible studies included clinical trials involving adult patients with AN or BN treated with rTMS. Data were pooled using fixed or random-effects models depending on heterogeneity. Mean differences (MD) or standardized mean differences (SMD) with 95% confidence intervals (CI) were calculated. Risk of bias was assessed using Cochrane RoB 2.0 and NIH tools.

**Results:**

Thirteen studies were included, yielding a total of n = 195 active rTMS treatments and n = 132 sham treatments. Compared to sham, rTMS did not significantly improve BMI in AN patients (MD: 0.19; 95% CI: -0.50, 0.88; P = 0.59). A significant moderate reduction in eating disorder severity was found in single-arm analysis (31 patients, SMD: -0.50, 95% CI [-0.90, -0.10], p = 0.01). Depression improved with rTMS over sham (105 patients, SMD: -0.41, 95% CI [-0.81, -0.02], p = 0.04), while anxiety, binge frequency, and vomiting frequency did not. Urge to eat increased in AN (30 patients, MD: 1.20, 95% CI [0.20, 2.21], p = 0.02) and decreased in BN (25 patients, MD: -10.25, 95% CI [-15.88, -4.62], p = 0.0004). No serious adverse events were reported.

**Conclusions:**

rTMS shows potential in improving eating disorder severity and depressive symptoms in AN, with disorder-specific modulation of food-related urges. Its effects on BMI and behavioral symptoms remain inconclusive. rTMS was well tolerated.

**Supplementary Information:**

The online version contains supplementary material available at 10.1186/s12991-026-00673-2.

## Introduction

Eating disorders are mental disorders that significantly impact psychosocial functioning and impair physical health. Key contributing factors include distorted attitudes toward weight, body shape, and food. The primary recognized disorders include anorexia nervosa (AN), bulimia nervosa (BN), binge eating disorder, and others [1]. A study showed that 14% of 56,901 visits to the emergency department were diagnosed with eating disorders [2]. Eating disorders affect people across all demographics, though adolescents, young adults, females, and individuals with concurrent depressive symptoms face heightened risk [3–6]. Furthermore, they impose a significant financial burden and greatly reduce patients’ health-related quality of life, with an estimated global loss of more than 3.3 million healthy lives [7, 8].

AN is a complex disorder marked by symptoms like extreme caloric restriction or behaviors leading to caloric elimination, intense fear of gaining weight, and disturbance in self-evaluation of body shape and weight, resulting in a notably low body mass index (BMI). AN has two subtypes, the restricting type and the binge-eating/purging type [9]. In the US, AN affects up to 4% of females and 0.3% of males [10]. Moreover, it carries a mortality rate of 5.1 per 1000, which is the highest among psychiatric disorders [11].

The pathophysiology of AN remains unclear, but recent research highlights structural and functional brain anomalies, including diminished gray matter in the cingulate cortex, occipital gyrus, and inferior parietal lobe (IPL), along with disrupted connectivity in several brain tracts [12–15]. Abnormal activity has been observed in brain areas related to body image perception, with correlations found between the drive for thinness and gray matter volume in the right IPL [16]. Patients with AN also show increased amygdala activation during body image-related tasks, which is linked to heightened emotional sensitivity and social comparison [15, 17]. Despite being categorized as a psychiatric functional disorder, AN has demonstrated genetic links, including its association with a specific chromosomal marker (rs4622308) [18, 19].

BN differs from AN in that individuals may have a normal or increased BMI. It involves episodes of binge eating characterized by consuming large amounts of food in a short period of time, accompanied by a loss of control over eating during the episode. These episodes are followed by inappropriate compensatory or purging behaviors, often through self-induced vomiting or laxative use [9]. Disrupted neural processing related to self-evaluation and body image has been associated with BN. Genetic, endocrine, environmental, and other comorbid factors are considered risk factors for BN [16]. Additionally, childhood sexual abuse is a strong risk factor for BN [20]. BN is more prevalent among females, with a lifetime prevalence of up to 3%, compared with 1% in males [10]. BN carries an annual mortality rate of 2.9 per 1000, with a significantly higher risk of death from unnatural causes than in individuals without BN [21].

The precise etiology of BN is unclear. Studies have shown that individuals with BN exhibit diminished activation in the frontostriatal area, which is crucial for decision-making, impulse control, and reward processing, contributing to the severity of BN symptoms. In addition, alterations in the brain’s reward systems occur in response to certain behaviors or environmental factors, reinforcing binge-eating/purge behavior [22]. Also, altered functional connectivity was observed between the thalamus and several regions, including the frontoparietal network, somatosensory network, visual network, and default mode network. These thalamic connectivity alterations were associated with the weekly frequency of binge-eating/purging episodes and external eating behavior scale scores [23].

Current treatment options include, but are not limited to, cognitive behavioral therapy (CBT) for AN, targeting cognitive, biological, and emotional factors that sustain disordered behaviors, in addition to pharmacologic treatments, including antidepressants, antipsychotics, and stimulants, especially for BN. However, these interventions are only moderately effective, with significant dropout and relapse rates following treatment, and around 20—30% of patients become refractory to treatment [24, 25]. Given the inefficiency of available therapies and the changes in brain activity and connectivity that underlie the pathophysiology of AN and BN [12, 23], brain-directed therapies that modulate brain activity may provide more effective interventions for AN and BN.

Repetitive transcranial magnetic stimulation (rTMS) is a non-invasive brain neuromodulation device. Thus, rTMS has fewer complications compared to deep brain stimulation (DBS) and is also suitable for patients who cannot undergo invasive procedures, and is more readily accepted by patients [26]. rTMS implies the repeated application of TMS pulses, allowing for sustained modulation of brain activity for longer periods. The specific effects of rTMS are influenced by various stimulation parameters, including intensity, frequency, and duration of treatment. This approach is generally well-tolerated and can be delivered in several formats: as a single pulse lasting milliseconds, paired pulses, or repeated trains extending from several seconds to minutes, depending on the intended therapeutic outcome [27]. Current rTMS protocols include low-frequency stimulation, which decreases cortical excitability through long-term depression (LTD); high-frequency stimulation, which increases cortical excitability through long-term potentiation (LTP); and theta-burst stimulation (TBS), which simulates endogenous brain rhythms. TBS can be administered intermittently (iTBS) for an excitatory effect or continuously (cTBS) for an inhibitory effect [28]. Although TBS is a modified version of rTMS, there is only one published protocol regarding its use in patients with persistent AN [29].

rTMS was investigated in psychiatric disorders such as depression and anxiety. rTMS is a licensed, safe, and effective method for treating depression, specifically for treating therapy-resistant depression in adults (APA guideline) [30]. rTMS also emerged as a promising treatment option for eating disorders such as BN and AN [31]. The possible mechanism of action of rTMS in AN and BN is attributed to neuromodulation of pathways related to self-control over disease symptoms, decreasing stress via decreasing cortisol levels, and inhibition of the amygdala [31, 32].

Some rTMS interventions have been performed in recent years regarding AN and BN. For AN, rTMS showed initial benefits in mood, quality of life, and dietary choices, in addition to BMI improvement, which was linked to changes in regional blood flow within the amygdala [33, 34]. For BN, a study found no significant difference between rTMS and sham treatments [35]. However, a related study found post-treatment gains in inhibitory control and decision-making, though clinical symptoms did not improve, suggesting rTMS may enhance neurocognitive functioning that could precede clinical benefits [36].

Published reviews investigating different neuromodulation techniques found that rTMS has little effect on psychological outcomes, with no effect on BMI in patients with EDs, specifically AN and BN [37]. Another review proposed possible efficacy; however, [38] limitations of these reviews include a small number of studies, small sample sizes, significant heterogeneity, failure to include all recently published trials, and the absence of a meta-analytic approach. Also published reviews included multiple neuromodulation techniques and diseases such as obesity, which is not considered an ED, and BEDs are more related to obesity due to the nature of impulsivity [39, 40]. We made the research question more specific to be more informative about the results. Due to the mixed results in the current literature and limitations of published reviews, this systematic review and meta-analysis aims to comprehensively summarize the existing evidence and provide an updated pooled analysis of findings from all published clinical trials on the use of rTMS for AN and BN. We focused on AN and BN with a single intervention to be more specific and answer one question, especially about the core of EDs (AN and BN). By doing so, we aim to offer clinically relevant practice guidance and inform future research directions.

## Methods

### Protocol documentation

This systematic review and meta-analysis was conducted according to the Preferred Reporting Items for Systematic Reviews and Meta-Analyses (PRISMA) guidelines (Supplementary Table 4) [41]. As it included published data, this study did not require ethical approval. It is registered with the International Prospective Register of Systematic Reviews (PROSPERO) under the identifier CRD42024594688.

### Search strategy

Two independent authors (Y.H and W.H) searched the following electronic databases: PubMed, Scopus, Web of Science (WOS), and Cochrane CENTRAL, from inception until May 5, 2025, using the following search strategy: (("repetitive transcranial magnetic stimulation" OR "transcranial magnetic stimulation" OR “rTMS”) AND (“eating disorders” OR “anorexia” OR “bulimia” OR “binge eating disorder”)). A tailored search strategy was applied to each database (Supplementary Table 1).

### Eligibility criteria

The inclusion criteria are as follows: (a) *Study design*: all clinical trials, randomized and non-randomized; (b) *Patients*: adults ≥ 18 years diagnosed with eating disorders, AN or BN; (c) *Intervention*: rTMS; (d) *Comparator*: sham rTMS or no comparator; and (e) *Outcomes*: BMI, depression, anxiety, change in binge-eating frequency, change in urge to eat/craving, and improvement in eating disorder scales.

The exclusion criteria included (a) observational studies, case reports, case series, editorials, narrative reviews, systematic reviews, meta-analyses, letters, abstracts, and commentaries; (b) adults younger than 18 years with a diagnosis of eating disorder; (c) interventions other than rTMS; and (d) animal/in vitro studies.

### Study selection

Using the search strategy, articles retrieved from various databases were imported into Rayyan [42] for screening by two independent authors. After removing the duplicates, an initial title and abstract screening was completed. Articles retrieved for full-text screening were thoroughly assessed based on the pre-defined eligibility criteria. An independent third author resolved any conflict during the process of screening.

### Data collection process and data items

Two authors independently extracted data from the included articles into a pilot-tested data extraction sheet. An independent investigator resolved any discrepancies that arose during the process of data collection. Data items that were extracted included:

- (a) *Study characteristics*: study ID, country, population, intervention, comparator, duration of outcome assessment, outcomes, and main findings.
- (b) *Baseline characteristics*: sample size, age, follow-up duration, number of sessions, BMI, gender, and duration of illness.
- (c) Outcome variables were *i) BMI* measured in kg/m^2^; *ii) Depression* assessed using the Montgomery–Åsberg Depression Rating Scale (MADRS), the Beck Depression Inventory (BDI), the depression subscale of the Hospital Anxiety and Depression Scale (HADS), the Hamilton Depression Rating Scale (HDRS), and the depression subscale of the Depression Anxiety Stress Scales-21 (DASS-21); *iii) Anxiety* measured using the anxiety subscale of the HADS, the anxiety subscale of the DASS-21, the Visual Analog Scale (VAS), and the Food and Fear of Missing Out (FoFM) Anxiety About Eating score; *iv) Changes in vomiting and binge eating frequency*; *v) Urges to eat/craving* were evaluated using the VAS, the Food Craving Questionnaire (FCQ), and the state version of the Food Craving Questionnaire (FCQ-S); *vi) Improvements in eating disorder symptoms* were assessed using the global score of the Eating Disorder Examination Questionnaire (EDE-Q Global) and the Eating Disorder Inventory-2 (EDI-2).

### Risk of bias assessment

The revised Cochrane Risk of Bias Tool (RoB 2.0) was used for the risk of bias assessment of randomized clinical trials (RCTs) [43]. All included RCTs were evaluated on five domains, namely bias from the randomization process, bias due to deviations from intended interventions, bias due to missing outcome data, bias in measurement of the outcomes, and bias in selection of the reported result. For single-arm trials, we used the National Institutes of Health tool (NIH), which consists of 12 questions, and the final decision was poor, fair, or good quality [44]. Any disagreement during the assessment of the risk of bias was resolved by discussion between the two authors.

### Statistical analysis

We used the RevMan (version 5.3; Copenhagen: The Nordic Cochrane Centre, The Cochrane Collaboration, 2014) tool for statistical analysis. We analyzed the pooled mean difference (MD) or standardized mean difference (SMD), both with a 95% confidence interval (CI). We used SMD only with outcomes that had different scales according to Cochrane guidelines [45]. Data reported as median and interquartile range (IQR) were transformed into mean and standard deviation (SD) using the Meta-Analysis Accelerator tool [46].

Our analysis involved two approaches. First, for the outcomes including both single- and double-arm studies, we did a single-arm meta-analysis for the rTMS group, pooling the change from baseline. Second, we did separate analyses for double-arm clinical trials, analyzing the pooled MD of the change from baseline between rTMS and sham groups. Regarding the latter one, we chose to analyze the change from baseline, not the post-treatment values, since this approach is better and more accurate according to Cochrane guidelines, as it controls possible differences at baseline between rTMS and sham groups [47]. We calculated the change from baseline using the Meta-Analysis Accelerator tool with a correlation coefficient of 0.5 [46]. We carried out an analysis of the outcomes reported in at least two studies. Forest plots were created for the visual assessment of pooled results. Outcomes were pooled using the fixed-effect model if there was no significant heterogeneity; otherwise, we shifted to the random-effects model. We performed Cochrane’s *χ*^2^ test to assess between-study heterogeneity and quantified heterogeneity across studies using Higgins *I*^2^ statistics, with a P-value < 0.1 or *I*^2^ > 50% considered significant heterogeneity. In this case, a leave-one-out analysis approach was employed to understand the impact of the exclusion of some studies on the overall pooled effect estimate. This analysis aimed to test the robustness of the meta-analysis findings by examining how the pooled effect estimate would change when some studies were systematically excluded. We didn’t test for publication bias, as none of our included outcomes were reported by at least ten studies [48]. Additionally, subgroup analyses were conducted based on eating disorder type (AN or BN), stimulation target, and the number of rTMS sessions, when applicable.

## Results

### Study selection

**Fig. 1:**
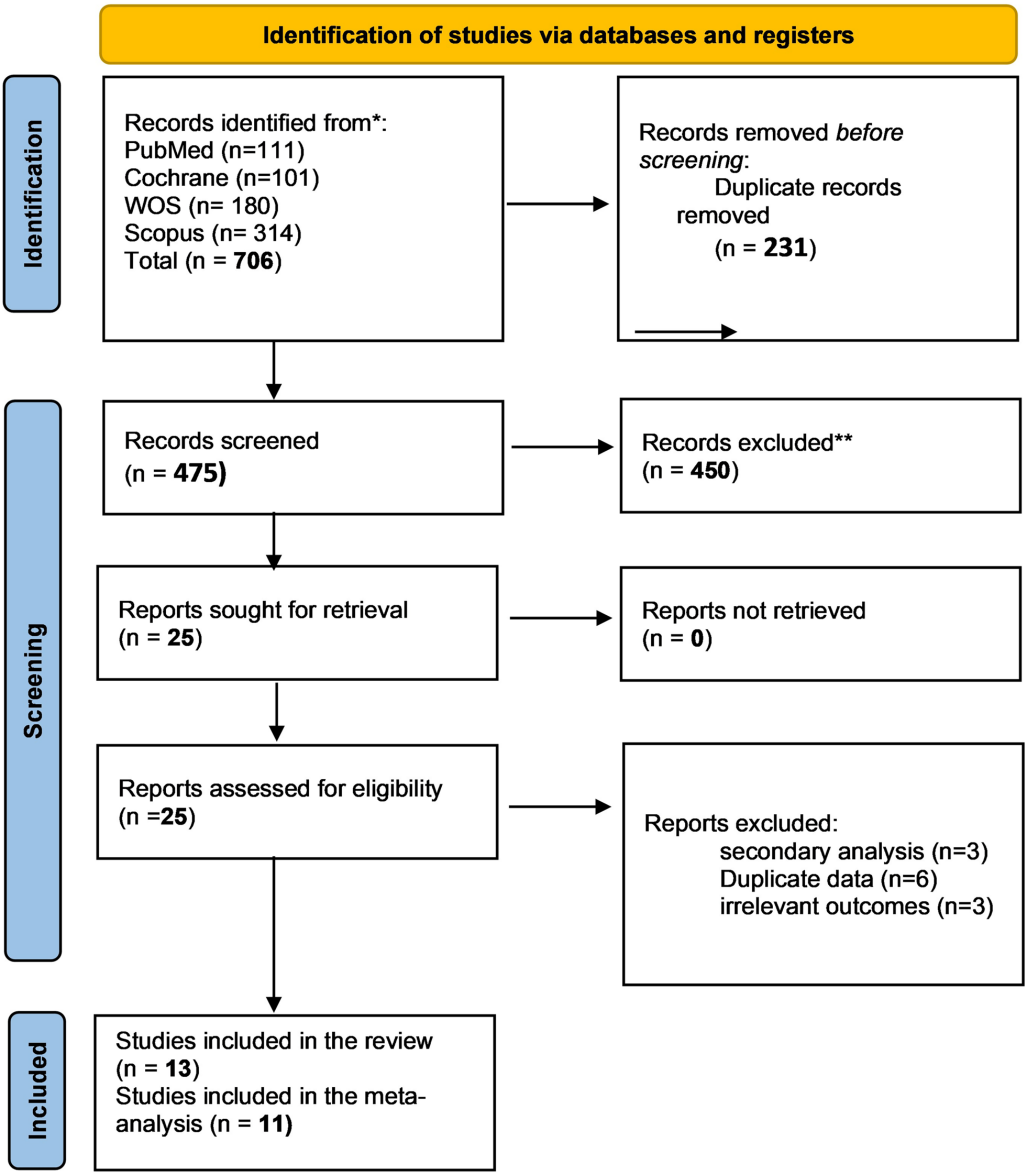
PRISMA flow diagram of study selection process. This figure outlines the identification, screening, eligibility, and inclusion stages of the systematic review, following the PRISMA 2020 guidelines. It shows the total records identified (n = 706), screened (n = 475), assessed for eligibility (n = 25), and final studies included (n = 13).

After database screening, 706 records were identified. Following the removal of duplicates, 475 records remained and were screened by title and abstract based on the inclusion criteria. A total of 25 records were selected for full-text retrieval and screening. After this process, 13 studies were included in the review. Of these, 11 were included in the analysis. *Woodside *et al*.* [49] was excluded from the analysis because it reported only pre- and post-treatment means without additional data. *Dalton *et al*. (2020)* [50] was also excluded due to potential patient overlap with *Dalton *et al*. (2018)* [33] (see Fig. 1).

### Characteristics of included studies

All of the included studies were clinical trials, with a total of 195 participants in the rTMS group (173 with AN or BN, excluding other eating disorders) and 132 participants in the sham group (118 with AN or BN, excluding other eating disorders). Eight studies are parallel sham-controlled randomized trials [33, 50, 35, 51–55], one study is a crossover sham-controlled randomized trial [56], and four studies are single-arm trials [49, 57–59]. Of the 13 included trials, seven trials were conducted on patients with AN, and six trials were conducted on BN patients. Our included trials were implemented in different countries, including the UK, Canada, France, the US, and Australia. The intervention of included studies was rTMS, mainly targeting the left dorsolateral prefrontal cortex (DLPFC) region. However, Muratore [56] targeted the right DLPFC, Chastan [52] targeted the right IPL (rIPL), and Blake Woodside [49] and Dunlop [59] targeted the dorsomedial prefrontal cortex (DMPFC). The number of sessions in the intervention group ranged from one session to a maximum of 30 sessions. The weighted mean age of the rTMS group was 28.96 years, and that of the sham group was 29.2 years, with a female predominance observed in all study samples for both groups. The studies used different tools and outcomes for the measurement of rTMS efficacy, such as BMI, improvement in eating disorder scales, including levels of depression and anxiety with different scales, number of binge episodes, urge to eat using VAS, cortisol concentration, and self-image regarding the shape. The characteristics of the included studies and their patients are shown in Tables 1 and 2.

**Table 1:** Summary of included studies

Study ID	Study design	Country	Population	Intervention	Comparator	Duration of outcome assessment	Outcomes	Main findings
Chastan 2024	Randomized sham-controlled study	France	Adult patients diagnosed with AN of the restricting subtype	10 Hz rTMS of the rIPL	Sham rTMS	3 months	Body Shape Questionnaire (BSQ), eating disorder symptoms, BMI, mood, anxiety, and safety	Real rTMS failed to make statistically significant improvements in body image distortion, or other related AN symptoms. Severe adverse events were observed across patient groups, primarily due to AN severity, with a good tolerance of the rTMS protocol
Blake Woodside 2021	Non-randomized open-label trial	Canada	Adult female patients with AN	10 Hz bilateral DMPFC-rTMS	N/A	4 weeks	Eating Disorders Examination (EDE 16.0D, the Beck Depression Inventory (BDI), the Hamilton Depression Inventory (HAM-D), the Beck Anxiety Inventory, and the Difficulties with Emotional Regulation Scale (DERS)	Significant improvements were seen in core AN pathology on the EDE global scale and, to a lesser extent, on the shape and weight concerns subscales. Significant improvements in comorbid anxiety and to a lesser extent, depression
Muratore 2021	Cross-over randomized trial	USA (New York)	Adult patients with AN	HF-rTMS (10 pulses/s, 4—s trains, 120% MT, 3000 pulses) to a targeted region of the right DLPFC	Sham rTMS	Post-intervention	Mean ratings of healthiness and tastiness, the proportion of trials on which the high-fat food was chosen over the reference item, and choices that reflect self-control	HF-rTMS was associated with increased ratings of healthiness for both low- and high-fat foods. Individuals also selected a significantly greater proportion of high-fat foods relative to a neutrally rated reference item
Dalton 2020	Randomized sham-controlled studyRandomized controlled study	UK	Women (≥ 18 years) with SE-AN	10 Hz High-frequency rTMS (110% MT, 1000 pulses) to the left DLPFC	Sham rTMS	4 months	Healthiness and Tastiness, food choice and self-control, Food choices by fat content, EDE-Q Global, DASS-21 Anxiety	Real rTMS reduced self-control use in the food choice task compared to baseline. However, it did not change food choice by fat content, nor did it change the rating of healthiness and tastiness
Dalton 2018	Randomized sham-controlled study	UK	Adult participants with a diagnosis of AN and a BMI > 14 kg/m2	10 Hz High-frequency rTMS (110% MT, 1000 pulses) to the left DLPFC in addition to treatment-as-usual	Sham rTMS	4 months	Recruitment, attendance, and retention rates, BMI, and eating disorder	rTMS was well tolerated with superior efficacy compared to sham over BMI, but a small effect, and symptoms of eating disorders. and moderate ot large effect on quality of life and mood
Gay 2016	Randomized sham-controlled study	France	Adult women with BN	10 Hz high-frequency rTMS program (20 trains of 5 s with 55-s intervals) targeting the left DLPFC	Sham rTMS	N/A	Number of vomiting episodes before the final visit, the mood at the final visit, and the modification in the outcomes before and after the baseline and final assessments, and safety	High frequency rTMS to the left DLPFC in patients with bulimic symptoms treated by adequate doses of SSRI does not yield greater benefits than a placebo. Results also suggest that rTMS is safe and well-tolerated in these patients
McClelland 2016	Randomized sham-controlled study	Australia	Adult patients with AN	10 Hz rTMS (110% MT, 1000 pulses over 20 min) to the left DLPFC	Sham rTMS	Post-intervention, 24 h	Core AN symptom (computed by summation of VAS scores on the urge to restrict, levels of feeling full, and levels of feeling fat; a maximum score of 30), Salivary cortisol concentrations, measures of psychopathology (e.g., mood), temporal discounting, Safety, tolerability, and acceptability	Individuals who received real rTMS (versus sham) tended to report reduced AN symptoms (statistical trend). However, given some improvements across this and other measures over time following both real and sham rTMS, there is also an indication of a placebo effect. The rate of TD was reduced following real (but not sham) rTMS (again, only at the trend level), suggesting that rTMS may encourage more prudent decision-making. Cortisol concentrations were not altered, and rTMS was a safe, tolerable, and acceptable procedure for people with AN
Dunlop 2015	Non-randomized open-label study	Canada	Adult patients with bingeing and purging behavior	10 Hz rTMS (120% MT, 5 s on, 10 s off, 3000 pulses) of theDMPFC	N/A	4 weeks	Weekly frequency of binge and purge episodes, the Eating Disorder Examination (EDE) response (defined as ≥ 50% decrease in objective binge (N1000 calories per binge) and purge episode frequency), Hamilton Rating Scale for Depression, Beck Depression Index-II (BDI-II), and Beck Anxiety Index (BAI)	Enhanced frontostriatal connectivity was associated with favorable outcomes in individuals receiving dmPFC-rTMS therapy for binge/purge behaviors. This treatment demonstrates potential benefits for some eating disorder patients with persistent binge and purge behaviors
Sutoh 2015	Non-randomized pilot study	Japan	Female subjects with BN	10 Hz rTMS (110% MT, 15 trains of 5 s with 55s intervals) on the left DLPFC	N/A	On the same day	Cerebral oxygenation change, food photo task, and a rock-paper-scissors task, hemoglobin concentration changes in the DLPFC with near-infrared spectroscopy during cognitive tasks measuring self-regulatory control in response to food photo stimuli, ratings for food cravings, Global Assessment of Functioning (GAF), Clinical Global Impression of disease severity, Hospital Anxiety and Depression Scale (HADS), and Global Assessment of Functioning (GAF)	Significant reductions in the subjective ratings for want to eat, urge to eat, and sense of hunger to the food stimuli, a significant decrease in cerebral oxygenation of the left
Van den Eynde 2013	Participants were informed that they would be randomized to sham or real rTMS; however, all received real rTMS	France	Ten people with a Diagnostic and Statistical Manual for Mental Disorders (DSM-IV-TR) diagnosis of AN (restricting and binge purging type	One session of real rTMS (10 Hz, 110% MT, 1000 pulses over 20 min) was delivered to the left DLPFC	N/A	Post-intervention, 3 weeks	urge to restrict, urge to exercise, feeling full, feeling fat, anxiety, safety and tolerability, Cortisol concentration, Eating Disorder Examination—Questionnaire (EDE-Q), Depression Anxiety Stress Scale (DASS)	Left DLPFC may reduce feelings of fullness, fatness, and anxiety in people with AN. The effect on more complex behaviors, such as the urge to restrict or the urge to exercise, is less clear. Clinically undesirable effects, such as reduced urge to eat, were not observed. One rTMS session did not alter subjective mood, tension, or hunger levels
Claudino 2010	Randomized sham-controlled study	England	Participants were a subsample Van den Eynde et al. 2010 (females BN)	One session of real rTMS (10 Hz, 110% MT, 1000 pulses) was delivered to the left DLPFC	Sham rTMS	N/A	Salivary cortisol concentration	Real rTMS reduced both salivary cortisol concentration and food craving more than sham rTMS
Van den Eynde 2010	Randomized sham-controlled study	England	Adult patients with BN with no specific type	20 Hz rTMS (120% MT, ten trains of 10 -s with a training interval of 60s) on the left DLPFC	Sham rTMS	24 h	Self-reported food craving immediately after the stimulation session and frequency of bingeing over a 24-h follow-up period, (VAS) of the urge to eat, VAS of hunger, VAS of tension, VAS of mood, VAS of the urge to binge eat, Food Craving Questionnaire-Trail	A single session of real rTMS reduces induced food cravings in people with bulimic disorder. In addition, a significant reduction was observed in bingeing in the 24 h after the real rTMS compared with the sham rTMS
Walpoth 2007	Randomized sham-controlled study	Austria	Women meeting DSM-IV criteria for BN	rTMS on the left DLPFC	Sham rTMS	1 month	Change in binges and purges, a decrease of the Hamilton Depression Rating Scale (HDRS), the Beck Depression Inventory (BDI), and the Yale-Brown Obsessive–Compulsive Scale	Results showed considerable improvements in binging and purging as well as depressive and obsessive–compulsive symptoms. However, no significant difference between the active treatment and the placebo-stimulated groups could be detected

AN: Anorexia nervosa; Hz: Hertz; MT: Motor threshold; SD: Standard Deviation; N/A: Data not available; HF-rTMS: High-frequency repetitive transcranial magnetic stimulation; DLPFC: Dorsolateral prefrontal cortex; MOCA: Montreal Cognitive Assessment Scale; BMI: Bodey Mass Index; SE-AN: Severe, enduring anorexia nervosa; EDE-Q: Eating Disorder Examination—Questionnaire; DASS: Depression Anxiety Stress scale; rIPL: right inferior parietal lobe; EDE: Eating Disorders Examination; BDI: Beck Depression Inventory; HAM-D: Hamilton Depression Inventory; BAI: the Beck Anxiety Inventory; DERS: Difficulties with Emotional Regulation Scale; BN: Bulimia nervosa; HDRS: Hamilton Depression Rating Scale; BDI-II: Beck Depression Index-II; HADS: Hospital Anxiety and Depression Scale; GAF: Global Assessment of Functioning; DMPFC: Dorsomedial prefrontal cortex

**Table 2:** Baseline Characteristics of included studies

Study ID	Sample size		Age, mean (SD)		Follow-up duration		Number of sessions		BMI, mean (SD)		Sex, female n (%)		Duration of illness, years [mean (SD)]	
	rTMS	Sham	rTMS	Sham	rTMS	Sham	rTMS	Sham	rTMS	Sham	rTMS	Sham	rTMS	Sham
Chastan 2024	7	10	35 (2.784)	30.25 (2.91)	3 months and 2 weeks	N/A	10	10	13.975 (0.640)	16.425 (0.485)	N/A	N/A	12 (3.340)	
Blake Woodside 2021	19	N/A	31.2 (9.8)	N/A	5 months	N/A	Range: 20–30	N/A	16.4 (1.3)	N/A	19 (100%)	N/A	N/A	N/A
Muratore 2021	12	4	30.7 (7.4)		N/A	N/A	1	1	18.2 (1.9)	18.3 (2.3)	12 (100%)		12.6 (9.5)	
Dalton 2020	16	14	N/A	N/A	4 months	4 months	20	20	15.90 (1.40)		N/A	N/A	15.70 (11.60)	
Dalton 2018	16	14	28.47 (9.48)	31.00 (11.29)	4 months	4 months	20	20	15.76 (1.62)	16.26 (1.22)	N/A	N/A	13.74 (10.74)	14.41 (11.09)
Gay 2016	23	24	27.75 (4.925)	29.5 (5.393)	2 weeks	2 weeks	10	10	N/A	N/A	23 (100%)	24(100%)	6 (9.87)	6 (9.87)
McClelland 2016	21	28	25.29 (6.88)	27.68 (9.89)	7 years	10 years	1	1	16.73 (1.69)	16.38 (1.67)	N/A	N/A	9.05 (7.02)	11.27 (8.01)
Dunlop 2015	28 (AN-BP [n = 11] BN [n = 17])	N/A	31.04 (9.48)	N/A	8 weeks	N/A	Mean = 21.2 (3.7), range = 18–30	N/A	19.03 (5.33)	N/A	26 (92.85%)	N/A	14.75 (10.19)	N/A
Sutoh 2015	8	N/A	24.8 (7.18)	N/A	1 week	N/A	1	N/A	19.54 (4.36)	N/A	8 (100%)	N/A	N/A	N/A
Van den Eynde 2013	10	N/A	28 (8.405)	N/A	N/A	N/A	1	N/A	15.75 (1.293)	N/A	N/A	N/A	N/A	N/A
Claudino 2010	11 [BN n = 7 EDNOS n = 4]	11 [BN n = 7 EDNOS n = 4]	28.2 (9.2)	28.9 (8.5)	90 min	90 min	1	1	26.8 (13.2)	22.2 (3.1)	11 (100%)	11 (100%)	(0–5 years, n = 4), (5–10 years, n = 2), (10–15 years, n = 3), (> 15 years n = 2)	(0–5 years, n = 7), (5–10 years n = 2), (10–15 years n = 1), (> 15 years, n = 1)
Van den Eynde 2010	17 [BN (n = 10) EDNOS (n = 7)]	20 [BN (n = 10) EDNOS (n = 10)]	30.5 (11.2)	29.5 (8.4)	24 h	24 h	1	1	25.8 (11.5)	25.0 (8.5)	14 (82.35%)	18 (90%)	(0–5 years: n = 6), (5–10 years: n = 5), (10–15 years: n = 3), (> 15 years: n = 3)	(0–5 years: n = 12), (5–10 years: n = 2), (10–15 years: n = 4), (> 15 years: n = 2)
Walpoth 2007	7	7	27.4 (4.8)	22.6 (2.6)	3 weeks	3 weeks	15	15	19.6 (2.4)	19.7 (1.7)	7 (100%)	7 (100%)	8.4 (3.2)	8.0 (2.8)

AN: Anorexia nervosa; SD: Standard Deviation; N/A: Data not available; BN: Bulimia nervosa; SSRIs: Selective serotonin reuptake inhibitors; SNRIs: Serotonin and norepinephrine reuptake inhibitors

### Risk of bias assessment

According to the ROB 2.0 tool for RCTs, six studies were rated as having *some concerns* [33, 35, 50, 52, 53, 56], while three were judged to be at *low risk* of bias [51, 54, 55]. Based on the NIH quality assessment tool, two single-arm trials were rated as *good quality* [57, 59] and two as *fair quality* [49, 58] (see Supplementary Tables 2 and 3).

### Outcomes

#### BMI (kg/m^2^)

**Fig. 2:**
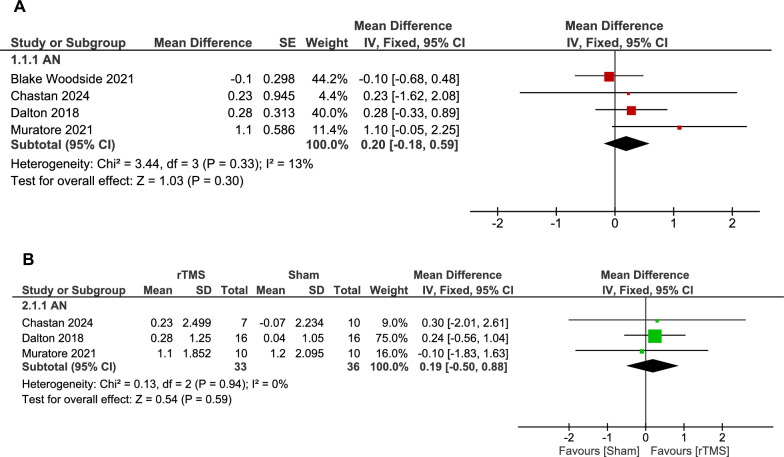
**A.** Forest plot: ffect of rTMS vs. sham on BMI in AN. *Comparison of BMI outcomes in AN patients between rTMS and sham groups showed no statistically significant difference*. **B.** Forest plot: effect of rTMS on BMI in single-arm AN studies. *Pooled analysis of BMI changes in AN patients following rTMS showed a non-statistically significant increase*.

The analysis of the three double-arm studies comparing rTMS (multi-session: 10 to 20 sessions) to sham in AN patients showed that the overall mean difference between rTMS and sham groups did not favor either intervention (69 patients, MD: 0.19, CI [-0.50, 0.88], p = 0.59). The pooled studies were homogeneous (p = 0.94, I^2^ = 0%) (see Fig. 2B).

The pooled analysis of the effect estimates of the rTMS group showed an insignificant increase in BMI in AN patients compared to pre-rTMS (52 patients, MD: 0.20, 95% CI [-0.18, 0.59], p = 0.30). The pooled studies were homogenous (p = 0.33, I^2^ = 13%) (see Fig. 2A).

A subgroup analysis was conducted according to rTMS target location. No significant difference was observed between the DLPFC and rIPL targets (p = 0.98). Additionally, neither subgroup demonstrated a statistically significant increase in BMI (DLPFC: p = 0.32; rIPL: p = 0.81) (see Supplementary Fig.1).

#### Eating disorder severity

A total of three studies reported eating disorder severity assessed by two validated scales, EDEQ Global and EDI-2.

**Fig. 3:**
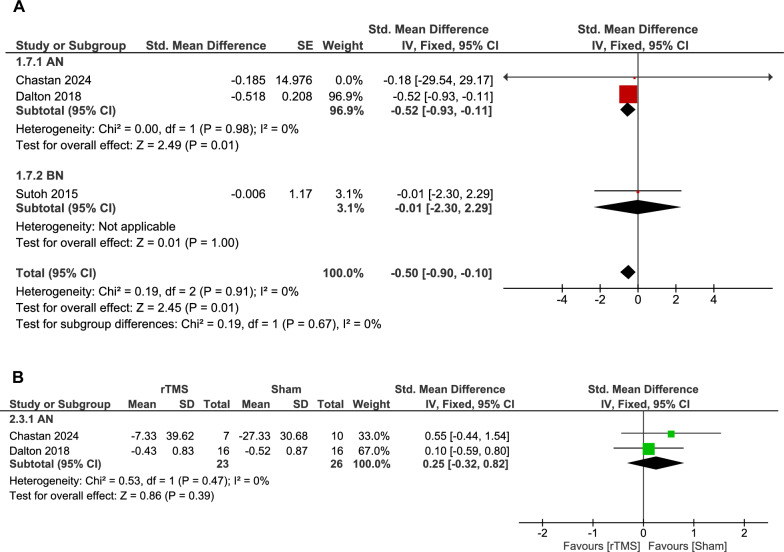
**A.** Forest plot: effect of rTMS on eating disorder severity – single-arm analysis. *Pooled analysis of standardized mean differences in eating disorder severity scores (e.g., EDE-Q Global, EDI-2) from baseline in patients undergoing rTMS showed a significant improvement.*** B.** Forest plot: effect of rTMS vs. sham on eating disorder severity in AN. *Comparison of eating disorder severity outcomes between rTMS and sham groups showed no statistically significant difference*.

We analyzed two AN double-arm trials comparing the rTMS group versus sham intervention, which did not favor either rTMS or sham (49 patients, SMD: 0.25, 95% CI [-0.32, 0.82], p = 0.39). No significant heterogeneity existed (p = 0.47, I^2^ = 0%) (Fig. 3B).

The pooled single-arm analysis for the rTMS group showed an overall moderate decrease in eating disorder severity compared to baseline (31 patients, SMD: -0.50, 95% CI [-0.90, -0.10], p = 0.01). Non-significant heterogeneity was found (p = 0.91, I^2^ = 0%) (Fig. 3A).

We performed a subgroup analysis for the rTMS group according to either AN or BN disorder type. We found that there was a significant moderate decrease in eating disorder severity in AN studies (SMD: -0.52, 95% CI [-0.93, -0.11], p = 0.01), while BN patients demonstrated no significant change in disease severity compared with pre-rTMS (SMD: -0.01, 95% CI [-2.30, 2.29], p = 1.0). The test for subgroup difference was insignificant (p = 0.67) (Fig. 3A).

Additional subgroup analysis based on rTMS target location demonstrated a significant moderate decrease in eating disorder severity in the DLPFC target only (SMD: -0.50, 95% CI [-0.90, -0.10], p = 0.01), whereas the rIPL target did not show significant improvement (SMD: -0.18, 95% CI [-29.54, 29.17], p = 0.99) compared with pre-rTMS. However, no significant difference was observed between the two target-location subgroups (p = 0.98) (Supplementary Fig. 2). Furthermore, when conducting subgroup analysis based on the number of rTMS sessions, there was a significant moderate decrease in eating disorder severity in the multiple-session subgroup (SMD: -0.52, 95% CI [-0.93, -0.11], p = 0.01), unlike the single-session subgroup, which did not show significant improvement compared with pre-rTMS (SMD: -0.01, 95% CI [-2.3, 2.29], p = 1.00). Similarly, no significant difference was observed between the two session-based subgroups (p = 0.67) (Supplementary Fig. 3).

#### Depression

A total of five studies were included in the analysis of the depression outcome. The analysis was performed for several validated scales, including DASS-2, HDRS, BDI, MADRS, and the depression subscale of the HADS.

**Fig. 4:**
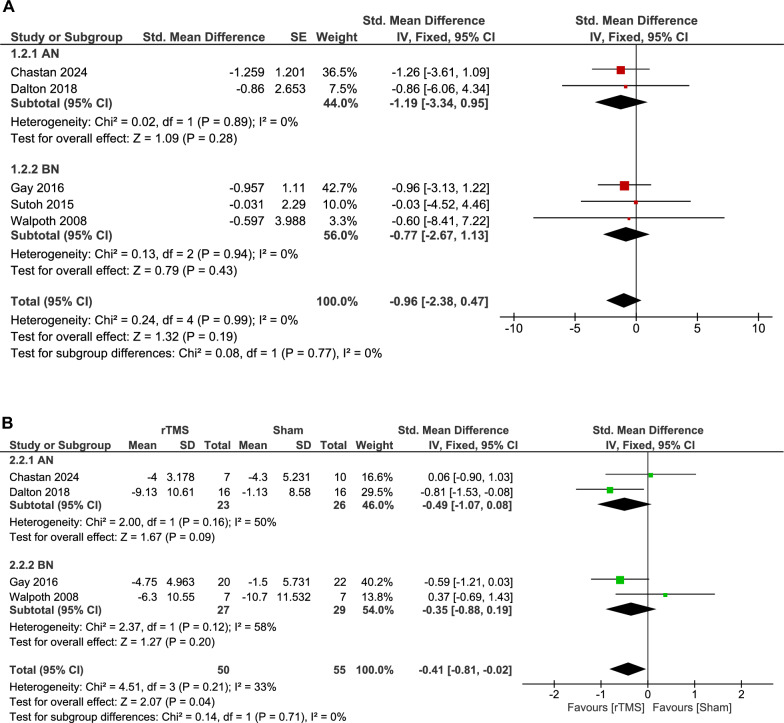
**A**. Forest plot: effect of rTMS on depression – single-arm analysis. *Pooled analysis of changes in depression scores pre- and post-rTMS using multiple validated scales showed no statistically significant effect*. **B**. Forest plot: effect of rTMS vs. sham on depression. *Comparison of standardized mean differences in depression outcomes across trials showed a statistically significant improvement in favor of rTMS over sham*.

The analysis of four double-arm studies reporting depression outcome favored the rTMS group over the sham group with a small, significant decrease (105 patients, SMD: -0.41, 95% CI [-0.81, -0.02], p = 0.04) with non-significant heterogeneity (p = 0.21, I^2^ = 33%) (Fig. 4B).

We performed subgroup analysis for double-arm studies according to disease type and found no significant difference between rTMS and sham interventions in either AN (p = 0.09) or BN (p = 0.2). The test for subgroup difference showed a non-significant difference between groups (p = 0.71) (Fig. 4B).

The analysis of the rTMS group showed insignificant change in depression scales compared to baseline (58 patients, SMD: -0.96, 95% CI [-2.38, 0.47], p = 0.19). Data were homogenous (p = 0.99, I^2^ = 0%) (Fig. 4A).

Subgroup analysis for the rTMS group was performed according to disease type. Non-significant improvement of depression following rTMS intervention in either the AN or BN subgroup (p = 0.28 and 0.43, respectively). The test for subgroup differences showed no significant difference between subgroups (p = 0.77) (Fig. 4A).

Additional subgroup analyses were conducted based on rTMS target location and number of sessions. Regarding target location, neither the DLPFC (p = 0.39) nor the rIPL (p = 0.29) showed a significant improvement in depression, and no significant difference was observed between the two subgroups (p = 0.75) (Supplementary Fig. 4). Similarly, subgroup analysis based on the number of sessions did not show a significant improvement in either single-session (p = 0.99) or multiple-session rTMS subgroups (p = 0.17), with no significant difference between these subgroups (p = 0.67) (Supplementary Fig. 5).

#### Anxiety

**Fig. 5:**
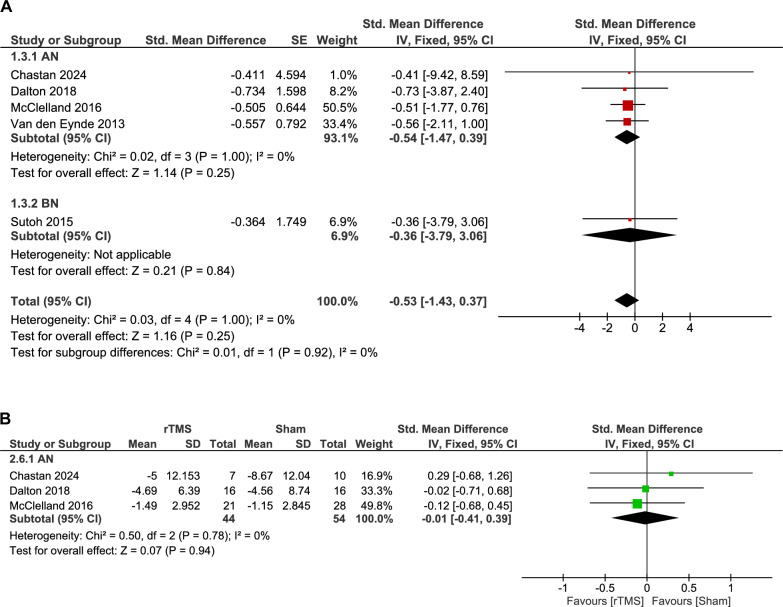
**A.** Forest plot: effect of rTMS on anxiety – single-arm analysis. *Pooled analysis of anxiety symptom changes following rTMS across studies using different scales showed no statistically significant improvement*. **B.** Forest plot: effect of rTMS vs. sham on anxiety. *Comparison of anxiety outcomes between rTMS and sham groups showed no statistically significant difference*.

Anxiety was reported in five studies with different assessment scales, including DASS-21, VAS anxiety, FoFM (anxiety about eating), HARS, and the anxiety subscale of the HADS. The analysis of three double-arm studies showed a non-significant difference between the rTMS group and sham intervention (98 patients, SMD: -0.01, 95% CI [-0.41, 0.39], p = 0.94), and no significant heterogeneity was found (p = 0.78, I^2^ = 0%) (Fig. 5B).

The pooled analysis of the rTMS group showed insignificant improvement in anxiety (61 patients, SMD: -0.53, 95% CI [-1.43, 0.37], p = 0.25). The pooled studies were homogenous (p = 1.00, I^2^ = 0%) (Fig. 5A).

Subgroup analysis was conducted according to disease type. Neither the AN nor the BN subgroups showed a significant improvement in anxiety, with p-values of 0.25 and 0.84, respectively. The test for subgroup difference showed a non-significant difference between subgroups (p = 0.92) (Fig. 5A).

Additional subgroup analyses were conducted based on rTMS target location and number of sessions. Regarding target location, Anxiety did not significantly improve with DLPFC stimulation (p = 0.25) or rIPL stimulation (p = 0.93), and there was no difference between the two targets (p = 0.98) (Supplementary Fig. 6). Similarly, subgroup analysis based on the number of sessions did not demonstrate a significant improvement in either single-session (p = 0.29) or multiple-session rTMS (p = 0.64), with no significant difference between these subgroups (p = 0.91) (Supplementary Fig. 7).

#### Change in binge eating frequency

Change in binge eating frequency was reported in three BN studies using different measurement approaches, including weekly binge frequency, binges per day, and the number of binge episodes in the last 15 days. Due to this variability in outcome reporting, we used the SMD for pooling the results. 

**Fig. 6:**
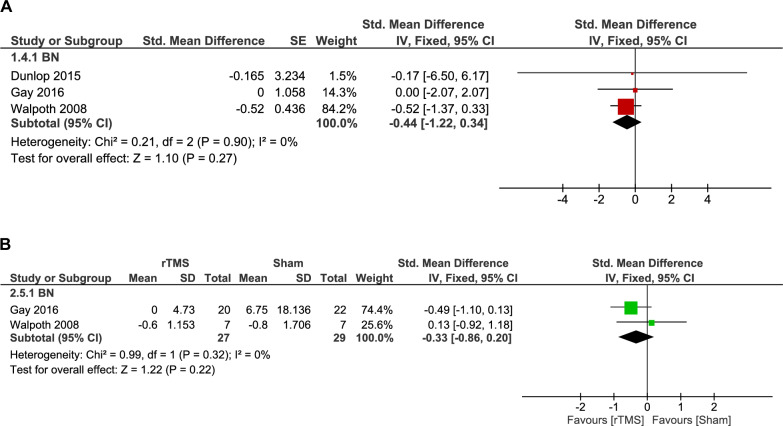
**A.** Forest plot: effect of rTMS on binge eating frequency in BN – single-arm analysis. (BN) *Pooled analysis of binge eating frequency changes in BN patients following rTMS showed no statistically significant effect*. **B.** Forest plot: effect of rTMS vs. sham on binge eating frequency in BN. *Comparison of binge eating frequency between rTMS and sham groups showed no statistically significant difference*.

The analysis of two double-arm studies showed no significant decrease in the frequency of binges (56 patients, SMD: -0.33, 95% CI [-0.86, 0.20], p = 0.22), and no heterogeneity was found (p = 0.32, I^2^ = 0%) (Fig. 6B).

The analysis of the rTMS group (multi-session: 10 to 30 sessions) showed a non-significant decrease in the frequency of binges (55 patients, SMD: -0.44, 95% CI [-1.22, 0.34], p = 0.27). The pooled studies were homogenous (p = 0.90, I^2^ = 0%) (Fig. 6A).

A subgroup analysis was conducted according to rTMS target location. No significant difference was observed between the DLPFC and DMPFC targets (p = 0.93). Additionally, neither subgroup demonstrated a statistically significant decrease in the frequency of binges (DLPFC: p = 0.27; DMPFC: p = 0.96) (Supplementary Fig. 8).

#### Change in urge to eat/craving

**Fig. 7:**
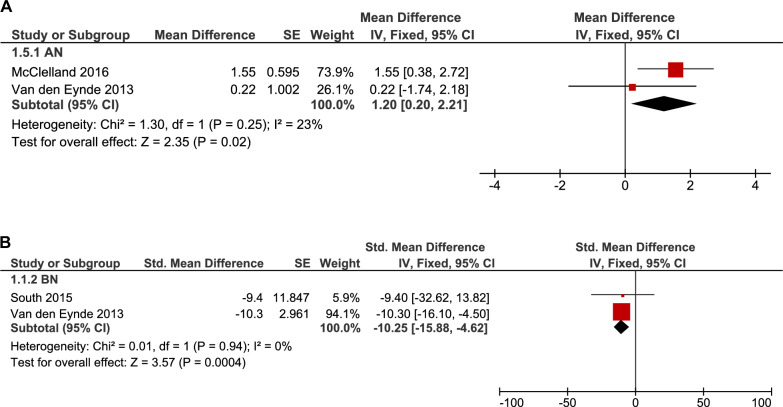
**A.** Forest plot: effect of rTMS on urge to eat in AN – single-arm analysis. *Pooled analysis of urge-to-eat scores in AN patients following rTMS showed a significant increase, suggesting modulation of appetite-related circuits*. **B.** Forest plot: effect of rTMS on urge to eat in BN – single-arm analysis. *Pooled analysis of urge-to-eat scores in BN patients following rTMS showed a significant reduction*

The urge-to-eat outcome was assessed using VAS, FCQ-S, and FCQ in four studies. The pooled analysis of the rTMS group (single session) in two AN studies showed a large significant increase in the urge-to-eat outcome (30 patients, MD: 1.20, 95% CI [0.20, 2.21], p = 0.02) with no significant heterogeneity (p = 0.25, I^2^ = 23%) (Fig. 7A), while the analysis of the rTMS group (single session) in two BN studies showed a large significant decrease in the urge to eat (25 patients, MD: -10.25, 95% CI [-15.88, -4.62], p = 0.0004), and no heterogeneity was found (p = 0.94, I^2^ = 0%) (Fig. 7B).

#### Vomiting frequency

**Fig. 8:**
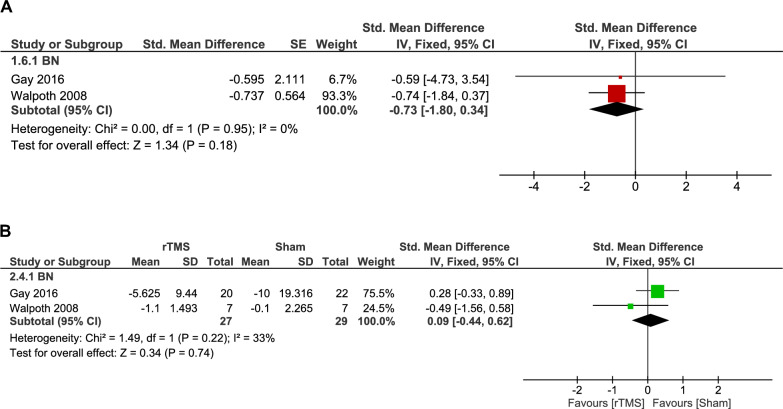
A. Forest plot: effect of rTMS on vomiting frequency in BN – single-arm analysis. *Pooled analysis of vomiting episode changes in BN patients following rTMS showed no statistically significant reduction*. B. Forest plot: effect of rTMS vs. sham on vomiting frequency in BN. *Comparison of rTMS and sham interventions in BN patients showed no statistically significant difference*.

Analysis of vomiting frequency in BN patients included a pooled analysis of two double-arm studies comparing rTMS to sham and showed no significant difference between groups (56 patients, SMD: 0.09, 95% CI [-0.44, 0.62], p = 0.74), with low heterogeneity (p = 0.22, I^2^ = 33%) (Fig. 8B).

Additionally, single-arm analysis of two studies, which used different measurement approaches, including the number of vomiting episodes in the last 15 days and vomiting frequency per day. Due to this variability, we used the SMD to pool the results. The single-arm analysis showed a non-significant decrease in vomiting episodes following rTMS (multi-session: 10 to 15 sessions, SMD: -0.73, 95% CI [-1.80, 0.34], p = 0.18), with no heterogeneity across studies (p = 0.95, I^2^ = 0%) (Fig. 8A).

#### Adverse effects

The majority of studies didn’t report adverse events, and no serious adverse events were reported. The most frequently reported adverse event is discomfort in the head.

## Discussion

Our systematic review and meta-analysis provides comprehensive qualitative and quantitative evidence on the efficacy of rTMS in managing eating disorders, with a focus on AN and BN. Our pooled analyses demonstrated mixed results across clinical and psychological outcomes. While rTMS did not significantly improve BMI in AN patients compared to sham controls, it was associated with a reduction in eating disorder severity in single-arm analyses, particularly among AN patients. Depression scores significantly improved in the rTMS group compared to the sham group, but anxiety outcomes did not differ across groups.

rTMS led to a significant increase in urge to eat in AN patients and a significant decrease in urge to eat in BN patients, suggesting potential disorder-specific modulation of appetite regulation. However, no significant reductions were observed in binge eating or vomiting frequency when comparing rTMS to sham in BN patients. Across the included studies, there were no reported serious adverse events. These findings partially support the primary objective of evaluating rTMS; it may be a potential neuromodulatory treatment beside the existing treatments for eating disorders; however, the results should be interpreted with caution due to certain limitations.

The analysis of sham-controlled and single-arm studies showed no effect on BMI; this can be due to severity, long duration of the disease, resistance to treatment, and very low BMI before treatment [52]. Also, ED severity outcome analysis showed no difference between the real rTMS group and the sham group. In contrast, the single-arm analysis of pre- and post-change showed a moderate decrease in disease severity. This effect is confounded by the placebo effect, but it suggests that there is a change in results over time, so long-course treatment may be beneficial for results to be evident. However, the AN patient subgroup compromised most of the effect and significance, while there was a single study in the BN subgroup, which is not enough to conclude the insignificance. So, more studies with a long treatment course are needed to conclude the efficacy of the intervention.

Change in binge eating frequency analysis showed no difference either in sham-controlled analysis or single-arm analysis; also, subgrouping according to disease type was insignificant. Along the same lines, rTMS showed no effect on vomiting frequency. This insignificance may be explained by the fact that these symptoms are not affected by brain modulation due to the neurobiology of the disease, which is not related to the inhibition pathway and is related to brain perception and flexibility [60]. Notably, a significant increase in the urge to eat was observed in AN patients following rTMS in single-arm analyses, suggesting a possible modulation of appetite-related neural pathways. Since it’s a desirable effect on those types of patients. It indicates the restoration of patients’ desire for eating and is an early symptom to be affected in the disease course. Contemporary to BN patients, who showed a significant decrease in urge to eat, which is also a desirable effect in BN patients. These findings suggest a potential role for rTMS in modulating food-related impulses despite the lack of broader clinical symptom improvements. But these results are based on two studies in each group, which need more trials to confirm.

Regarding psychological symptoms, rTMS shows a small decrease in the severity of depression in sham-controlled analysis, which is in line with the application and recommendation of this intervention in managing depression in previous literature [61, 62]. However, this effect wasn’t significant in each disease type subgroup, which may be related to the small number of studies and small sample size. These findings need more studies and a larger sample size to evaluate their efficacy. Additionally, rTMS was associated with no change in overall anxiety severity or in any subgroup; neither the number of sessions nor the target was associated with a significant change. However, the analysis was limited by a small sample size and underpowered studies [51].

Due to variability in protocols of included studies, we conducted another subgrouping according to brain targets and number of sessions whenever there were available numbers of studies. No significant differences were observed between subgroups in outcomes, including binge eating frequency, anxiety, BMI, or depression, whether based on the number of sessions (single vs. multiple) or the brain target. However, ED severity outcome showed significant improvement with DLPFC target only and with multiple sessions compared to a single session. These findings regarding the brain target are in line with findings of altered connectivity between the DLPFC and striatum found in AN patients [63]. Future study protocols might consider targeting the DLPFC and applying multiple sessions instead of a single session. However, these results should be interpreted with caution due to the limited number of studies in some subgroups and heterogeneity in stimulation parameters and follow-up durations. 

Regarding body image perception, Dalton et al. and Woodside et al. found sustained improvements in fullness and body image perception and modest improvements regarding weight and shape [33]. However, Chastan et al. [52] investigated body shape perception in patients with restricting AN. After 10 sessions of active rTMS, they did not find any significant improvements in body shape perception. This could be attributed to the severity of symptoms [e.g., significant body image distortion and low BMI < 14], long disease duration, and refractoriness to previous interventions [52].

Notably, Dunlop et al. [59] highlighted inter-individual variability in response to rTMS and pointed out personalized treatment based on the special functional connectivity state for each patient at baseline. They found that increased response rate was associated with low pretreatment functional connectivity, which significantly increased after treatment, compared to non-responding patients who showed no or decreased change after treatment [59]. This should be addressed in further studies to prove this association and determine the group of patients who would benefit most from rTMS.

Despite the insignificant results in most of the BN studies, they observed changes in the hypothalamic–pituitary–adrenal axis [HPAA] activity [55], neuropsychological performance [36], cerebral oxygenation [58], and cue-induced food craving [54] due to the modest effect observed on the DLPFC.

While rTMS shows promise in improving some neurocognitive and psychological symptoms, particularly in AN, its effects on core behavioral symptoms such as bingeing and purging remain inconclusive, warranting further high-quality trials with larger samples and standardized protocols. We focused exclusively on rTMS rather than other forms of neurostimulation, such as TBS, to keep our research question and conclusions specific and focused, as recommended by the Cochrane Handbook. Further studies are needed to evaluate other neurostimulation techniques in patients with AN.

### rTMS for AN

Functional magnetic resonance imaging (fMRI) studies reveal altered connectivity in neural circuits related to food-related decision-making, self-control, and emotional regulation in patients with AN [64]. Abnormal connectivity between the DLPFC and the striatum is noted, with enhanced connectivity when patients view low-fat food imagery and diminished connectivity with high-fat food imagery, contrasting with healthy controls. Disrupted connectivity between the ventral caudate and frontal areas in AN suggests that this network may contribute to maladaptive behaviors, such as restrictive eating and heightened self-control over food choices. However, findings on ventral frontal-striatal network connectivity are inconsistent [63, 65].

The possible mechanism of action of rTMS in patients with AN involves modulating neural circuits related to self-control, emotional regulation, and reward processing. rTMS helps recalibrate activity in this fronto-limbic network, potentially reducing maladaptive control and anxiety responses. rTMS was also found to reduce amygdala cerebral blood flow, which may lessen fear responses linked to eating [34]. In addition, rTMS reduces amygdala activity, which may correlate with clinical improvements [32, 66]. HF-rTMS has also been associated with DMPFC hyperactivity, facilitating more adaptive top-down control in AN binge-eating/purge subtypes [59].

### rTMS for BN

Functional brain imaging showed that patients with BN exhibit hypoactivity in the frontostriatal circuit, besides disrupted functioning in the striatum, insula, and prefrontal cortex. Conversely, hyperactivity was observed in the anterior cingulate cortex, orbitofrontal cortex, and parieto-occipital regions [67, 68].

The possible mechanism of action of rTMS in patients with BN involves the neuromodulatory effect. Treatment responders demonstrate improved functional connectivity, potentially enhancing control over binge and purge behaviors [64]. Also, rTMS modulates serotonin (5-HT) activity, often disrupted in BN [69, 70], aiding appetite regulation and reducing impulsivity, thereby decreasing binge eating and purging episodes [71]. Additionally, rTMS reduces pro-inflammatory cytokines and modulates microglial function linked to cognitive impairments and depression [23, 72]. Furthermore, rTMS promotes neuroplasticity by influencing brain-derived neurotrophic factor (BDNF) levels, which are important for neuronal adaptation and memory. Increased BDNF post-treatment correlates with symptom improvement in BN [73, 74]. Finally, rTMS can reduce HPAA activity, consequently reducing cortisol levels. This may mitigate stress-related increases in appetite, contributing to reduced binge-eating behaviors [31, 55].

### Adverse events

Some studies reported mild and insignificant symptoms, including transient headache, eye twitches, and scalp discomfort. Other adverse events among patients with AN were largely associated with the severity of AN symptoms, as in Chastan et al. [52]. Adverse effect monitoring and reporting were inconsistently detailed across studies, limiting a comprehensive understanding of the safety of rTMS for AN and BN.

### Clinical implications

Our findings suggest that rTMS may offer modest benefits in improving psychological symptoms associated with eating disorders, particularly eating disorder severity and depression, most notably in patients with AN. However, its effects on anxiety, binge eating, vomiting frequency, and BMI were not statistically significant compared to the sham. Importantly, rTMS did not demonstrate a significant impact on BMI in AN, where weight restoration is a critical therapeutic goal. These results indicate that while rTMS may not directly contribute to core behavioral or weight-related outcomes, it could serve as a valuable adjunct within a multidisciplinary treatment framework. Its primary utility may lie in enhancing emotional and cognitive domains of recovery, complementing established interventions focused on nutritional rehabilitation and medical stabilization. Future research should explore optimized stimulation parameters, longer treatment durations, and patient-specific factors to better harness the therapeutic potential of rTMS in eating disorders.

### Limitations and future directions

Studies on rTMS for eating disorders show substantial variability in treatment parameters, including the specific brain area targeted, stimulation intensity, session duration, and frequency. This inconsistency poses challenges for drawing clear conclusions and limits the ability to generalize findings. Standardizing rTMS protocols in future studies would greatly facilitate cross-study comparisons. Additionally, many studies have short follow-up periods, which may not fully capture the long-term effectiveness and safety of rTMS. Extended follow-up periods are needed to evaluate lasting effects better and identify any delayed adverse effects. Also, the predominance of female participants limits generalizability across genders. Future research should investigate rTMS effects across diverse demographic groups to enhance applicability.

Another limitation is the lack of reported information on concurrent treatments used alongside TMS, which prevents the understanding of whether medications might enhance or reduce the efficacy of rTMS. It is also essential to consider the duration of AN and BN in study subjects, as differences in illness duration could influence response variability. Such insights are crucial for optimizing stimulation parameters to suit individual patient needs. In addition, details on potential side effects, like cognitive or mood changes and long-term adverse effects, were often lacking. Consistent and detailed adverse effect monitoring is essential for patient safety and optimizing treatment protocols. Future research should establish standardized protocols for adverse effect tracking, encompassing both immediate and long-term outcomes. This would help identify potential risks and improve rTMS safety and tolerability in treating BN.

The impact of handedness on rTMS outcomes in AN and BN remains unclear due to inconsistent reporting. Another limitation is the small sample sizes in the included studies, which limits the statistical power and the ability to detect significant differences between active and sham rTMS groups. Future studies should include larger sample sizes to improve the reliability and validity of the findings. Additionally, the small number of included studies, with some subgroups including only one study and some outcomes including only two studies, further limits the strength of the evidence; therefore, these outcomes need to be reported and interpreted with caution. Moreover, the included studies involve diverse patient populations with different types of AN, varying ages, and different baseline neural circuitry. This heterogeneity makes it difficult to identify which specific subgroups benefit most from rTMS. Another limitation of our findings is the inclusion of studies rated as having some concerns in certain domains of the risk of bias assessment. Pre-treatment neuroimaging may play a crucial role in optimizing stimulation parameters for better outcomes. Future studies should focus on more homogeneous populations or conduct subgroup analyses to better understand the effects of rTMS in specific patient groups. Future studies also should consider pre-treatment functional connectivity in the application of rTMS, as Dunlop's [59] study found that decreased functional connectivity before treatment is a predictor of response.

Furthermore, while some studies propose mechanisms for the effects of rTMS, detailed investigations into how rTMS influences brain activity to alleviate eating disorder symptoms are lacking. The need to understand neurobiological changes and their relationship to clinical outcomes is warranted. Therefore, additional mechanistic studies are essential for deepening our understanding of the therapeutic potential of rTMS in eating disorders.

## Conclusion

This systematic review and meta-analysis provides a comprehensive evaluation of rTMS as a therapeutic intervention for AN and BN. While rTMS did not significantly improve BMI or core behavioral symptoms such as bingeing and purging compared to sham stimulation, it showed potential benefits in reducing eating disorder severity, particularly among patients with AN, decreasing depressive symptoms, and modulating craving. The effect on anxiety was inconclusive.

These findings highlight the potential role of rTMS as an adjunctive tool targeting the psychological and neurocognitive dimensions of eating disorders, rather than as a standalone intervention for physical or behavioral outcomes. Given the heterogeneity of protocols and the limited sample sizes in existing studies, further high-quality RCTs with standardized rTMS parameters, longer stimulation durations, and extended follow-up are needed to clarify its clinical utility. Future research should also explore personalized approaches that consider the distinct neurobiological and symptomatic profiles of AN and BN.

## Supplementary Information

Supplementary material 1.

## Data Availability

Data were publicly available.

## Declarations

### Generative AI in scientific writing

We declare that we have not used AI in writing any part of this abstract.
