# Supplementary material for: Efficacy and safety of repetitive transcranial magnetic stimulation in anorexia and bulimia nervosa: a systematic review and meta-analysis
Source: Ann Gen Psychiatry. 2026 Jun 1;25:46. doi: 10.1186/s12991-026-00673-2 (PMC13224673; doi:10.1186/s12991-026-00673-2)

**Supplementary Table 1: showing the search strategy applied to each database**

| Database 5/5/2025 | Search strategy |
| --- | --- |
| PubMed | (("Repetitive transcranial magnetic stimulation" OR "transcranial magnetic stimulation" OR “rTMS”) AND (“eating disorders” OR “anorexia” OR “bulimia” OR “binge eating disorder”)) |
| SCOPUS | ( ( "Repetitive transcranial magnetic stimulation" OR "transcranial magnetic stimulation" OR "rTMS" ) AND ( "eating disorders" OR "anorexia" OR "bulimia" OR "binge eating disorder" ) ) AND ( LIMIT-TO ( SUBJAREA , "MEDI" ) ) AND ( LIMIT-TO ( DOCTYPE , "ar" ) ) AND ( LIMIT-TO ( EXACTKEYWORD , "Human" ) ) |
| Web Of Science | (("Repetitive transcranial magnetic stimulation" OR "transcranial magnetic stimulation" OR “rTMS”) AND (“eating disorders” OR “anorexia” OR “bulimia” OR “binge eating disorder”)) in all fields |
| Cochrane | ("Repetitive transcranial magnetic stimulation" OR "transcranial magnetic stimulation" OR “rTMS”) in All Text AND (“eating disorders” OR “anorexia” OR “bulimia” OR “binge eating disorder”) in All Text  Clinical trial filter |

**Supplementary Table 2**. The risk of bias assessment table for randomized controlled trials using the RoB-2 tool

| **Study ID** | **D1** | **D2** | **D3** | **D4** | **D5** | **Overall** |
| --- | --- | --- | --- | --- | --- | --- |
| **Chastan 2024** | Some concerns | Low | Low | Low | Low | Some concerns |
| **Muratore 2021** | Low | Low | Some concerns | Low | Low | Some concerns |
| **Dalton 2020** | Some concerns | Low | Low | Low | Low | Some concerns |
| **Dalton 2018** | Some concerns | Low | Low | Low | Low | Some concerns |
| **Gay 2016** | Low | Some concerns | Low | Low | Low | Some concerns |
| **McClelland 2016** | Low | Low | Low | Low | Low | Low |
| **Claudino 2010** | Low | Low | Low | Low | Low | Low |
| **Van den Eynde 2010** | Low | Low | Low | Low | Low | Low |
| **Walpoth 2007** | Some concerns | Some concerns | Low | Low | Low | Some concerns |

**D1**: Bias due to the randomization process. **D2**: Bias due to deviation from the intended intervention. **D3**: Bias due to missing outcome data. **D4**: Bias in the measurement of outcomes. **D5**: Bias in the selection of the reported results

**Supplementary Table 3**. The risk of bias assessment of single-arm trials using the NIH tool

| **Domain** | **Blake Woodside 2021** | **Dunlop 2015** | **Sutoh 2015** | **Van den Eynde 2013** |
| --- | --- | --- | --- | --- |
| Was the research question or objective in this paper clearly stated? | 1 | 1 | 1 | 1 |
| Were eligibility/selection criteria for the study population prespecified and clearly described? | 1 | 1 | 1 | 1 |
| Were the participants in the study representative of those who would be eligible for  The test/service/intervention in the general or clinical population of interest? | 0 | 1 | 0 | 1 |
| Were all eligible participants who met the prespecified entry criteria enrolled? | 0 | 1 | 1 | 1 |
| Was the sample size sufficiently large to provide confidence in the findings? | 0 | 0 | 0 | 0 |
| Was the test/service/intervention clearly described and delivered consistently across the study population? | 1 | 1 | 1 | 1 |
| Were the outcome measures prespecified, clearly defined, valid, and reliable | 1 | 1 | 1 | 1 |
| Were the people assessing the outcomes blinded to the participants' exposures/interventions? | 0 | 0 | 0 | 0 |
| Was the loss to follow-up after baseline 20% or less? Were those lost to follow-up accounted for in the analysis | 1 | 1 | 1 | 1 |
| Were outcome measures of interest taken multiple times before the intervention | 1 | 1 | 1 | 1 |
| and multiple times after the intervention (i.e., did they use an interrupted time-series design)? | 1 | 1 | 1 | 1 |
| If the intervention was conducted at a group level (e.g., a whole hospital, a community, etc.), did the statistical analysis take into account the use of individual-level data to determine effects at the group level? | NA | NA | NA | NA |
| **Final score** | **66.6%** | **83.3%** | **75%** | **83.3%** |
| **Final assessment Quality Rating: Poor <50%, Fair 50–75%, Good ≥75%** | **fair** | **good** | **fair** | **good** |

**Supplementary Table 4.** PRISMA Checklist

| **Section and Topic** | **Item #** | **Checklist item** | **Location where item is reported** |
| --- | --- | --- | --- |
| **TITLE** | | |  |
| Title | 1 | Identify the report as a systematic review. | Page #1 |
| **ABSTRACT** | | |  |
| Abstract | 2 | See the PRISMA 2020 for Abstracts checklist. | Page #3 |
| **INTRODUCTION** | | |  |
| Rationale | 3 | Describe the rationale for the review in the context of existing knowledge. | Page #7 |
| Objectives | 4 | Provide an explicit statement of the objective(s) or question(s) the review addresses. | Page #7 |
| **METHODS** | | |  |
| Eligibility criteria | 5 | Specify the inclusion and exclusion criteria for the review and how studies were grouped for the syntheses. | Page #8 |
| Information sources | 6 | Specify all databases, registers, websites, organisations, reference lists and other sources searched or consulted to identify studies. Specify the date when each source was last searched or consulted. | Page #7 |
| Search strategy | 7 | Present the full search strategies for all databases, registers and websites, including any filters and limits used. | Page #7 and #8 |
| Selection process | 8 | Specify the methods used to decide whether a study met the inclusion criteria of the review, including how many reviewers screened each record and each report retrieved, whether they worked independently, and if applicable, details of automation tools used in the process. | Page #8 |
| Data collection process | 9 | Specify the methods used to collect data from reports, including how many reviewers collected data from each report, whether they worked independently, any processes for obtaining or confirming data from study investigators, and if applicable, details of automation tools used in the process. | Page #9 |
| Data items | 10a | List and define all outcomes for which data were sought. Specify whether all results that were compatible with each outcome domain in each study were sought (e.g. for all measures, time points, analyses), and if not, the methods used to decide which results to collect. | Page #9 |
|  | 10b | List and define all other variables for which data were sought (e.g. participant and intervention characteristics, funding sources). Describe any assumptions made about any missing or unclear information. | Page #9 |
| Study risk of bias assessment | 11 | Specify the methods used to assess risk of bias in the included studies, including details of the tool(s) used, how many reviewers assessed each study and whether they worked independently, and if applicable, details of automation tools used in the process. | Page #9 and #10 |
| Effect measures | 12 | Specify for each outcome the effect measure(s) (e.g. risk ratio, mean difference) used in the synthesis or presentation of results. | Page #10 and #11 |
| Synthesis methods | 13a | Describe the processes used to decide which studies were eligible for each synthesis (e.g. tabulating the study intervention characteristics and comparing against the planned groups for each synthesis (item #5)). | Page #8 |
|  | 13b | Describe any methods required to prepare the data for presentation or synthesis, such as handling of missing summary statistics, or data conversions. | Page #10 and #11 |
|  | 13c | Describe any methods used to tabulate or visually display results of individual studies and syntheses. | Page #10 and #11 |
|  | 13d | Describe any methods used to synthesize results and provide a rationale for the choice(s). If meta-analysis was performed, describe the model(s), method(s) to identify the presence and extent of statistical heterogeneity, and software package(s) used. | Page #10 and #11 |
|  | 13e | Describe any methods used to explore possible causes of heterogeneity among study results (e.g. subgroup analysis, meta-regression). | Page #10 and #11 |
|  | 13f | Describe any sensitivity analyses conducted to assess robustness of the synthesized results. | Page #10 and #11 |
| Reporting bias assessment | 14 | Describe any methods used to assess risk of bias due to missing results in a synthesis (arising from reporting biases). | Page #9 and #10 |
| Certainty assessment | 15 | Describe any methods used to assess certainty (or confidence) in the body of evidence for an outcome. | NA |
| **RESULTS** | | |  |
| Study selection | 16a | Describe the results of the search and selection process, from the number of records identified in the search to the number of studies included in the review, ideally using a flow diagram. | Page #11 |
|  | 16b | Cite studies that might appear to meet the inclusion criteria, but which were excluded, and explain why they were excluded. | Page #11 |
| Study characteristics | 17 | Cite each included study and present its characteristics. | Page #11 and #12 |
| Risk of bias in studies | 18 | Present assessments of risk of bias for each included study. | Page #12 |
| Results of individual studies | 19 | For all outcomes, present, for each study: (a) summary statistics for each group (where appropriate) and (b) an effect estimate and its precision (e.g. confidence/credible interval), ideally using structured tables or plots. | Page #13 to #16 |
| Results of syntheses | 20a | For each synthesis, briefly summarise the characteristics and risk of bias among contributing studies. | Page #13 to #16 |
|  | 20b | Present results of all statistical syntheses conducted. If meta-analysis was done, present for each the summary estimate and its precision (e.g. confidence/credible interval) and measures of statistical heterogeneity. If comparing groups, describe the direction of the effect. | Page #13 to #16 |
|  | 20c | Present results of all investigations of possible causes of heterogeneity among study results. | Page #13 to #16 |
|  | 20d | Present results of all sensitivity analyses conducted to assess the robustness of the synthesized results. | Page #16 |
| Reporting biases | 21 | Present assessments of risk of bias due to missing results (arising from reporting biases) for each synthesis assessed. | NA |
| Certainty of evidence | 22 | Present assessments of certainty (or confidence) in the body of evidence for each outcome assessed. | NA |
| **DISCUSSION** | | |  |
| Discussion | 23a | Provide a general interpretation of the results in the context of other evidence. | Page #17 to #23 |
|  | 23b | Discuss any limitations of the evidence included in the review. | Page # 23 and #24 |
|  | 23c | Discuss any limitations of the review processes used. | Page # 23 and #24 |
|  | 23d | Discuss implications of the results for practice, policy, and future research. | Pages # 23 |
| **OTHER INFORMATION** | | |  |
| Registration and protocol | 24a | Provide registration information for the review, including register name and registration number, or state that the review was not registered. | Page #7 |
|  | 24b | Indicate where the review protocol can be accessed, or state that a protocol was not prepared. | Protocol can be accessed |
|  | 24c | Describe and explain any amendments to information provided at registration or in the protocol. | Not reported |
| Support | 25 | Describe sources of financial or non-financial support for the review, and the role of the funders or sponsors in the review. | No fund |
| Competing interests | 26 | Declare any competing interests of review authors. | No conflict of interest |
| Availability of data, code and other materials | 27 | Report which of the following are publicly available and where they can be found: template data collection forms; data extracted from included studies; data used for all analyses; analytic code; any other materials used in the review. | Data publicly available |

**Supplementary Figure 1.** Subgroup Analysis Based on Target Location for the Effect of rTMS on BMI in Single-Arm AN Studies

*No statistically significant difference was observed between subgroups*


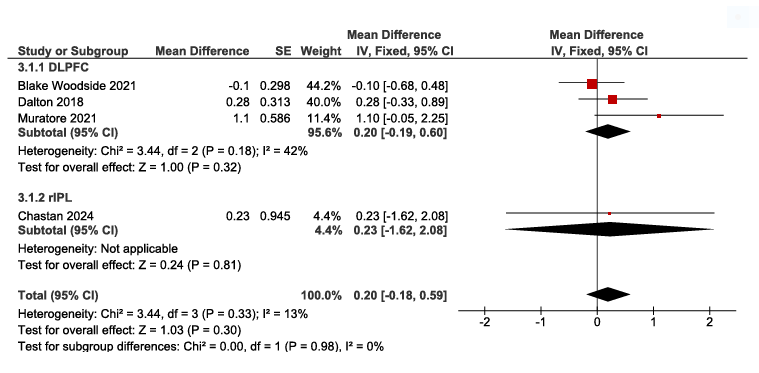


**Supplementary Figure 2.** Subgroup Analysis Based on Target Location for the Effect of rTMS on Eating Disorder Severity – Single-Arm Analysis

*No statistically significant difference was observed between subgroups*


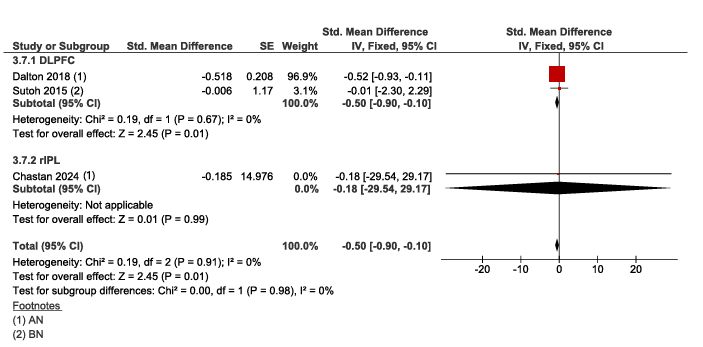


**Supplementary Figure 3.** Subgroup Analysis Based on Number of Sessions for the Effect of rTMS on Eating Disorder Severity – Single-Arm Analysis

*No statistically significant difference was observed between subgroups*


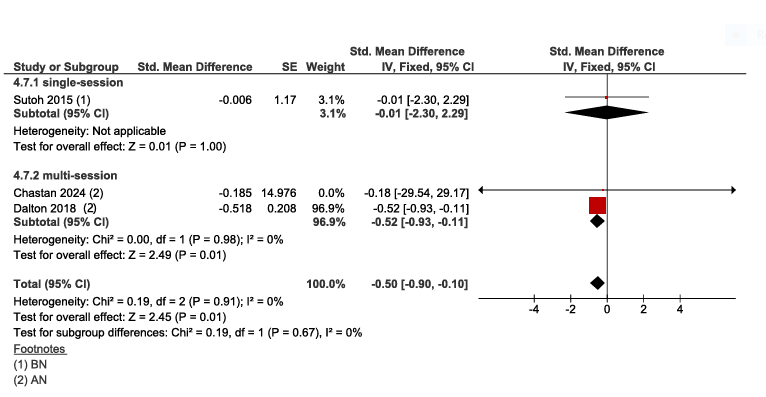


**Supplementary Figure 4.** Subgroup Analysis Based on Target Location for the Effect of rTMS on Depression – Single-Arm Analysis

*No statistically significant difference was observed between subgroups*


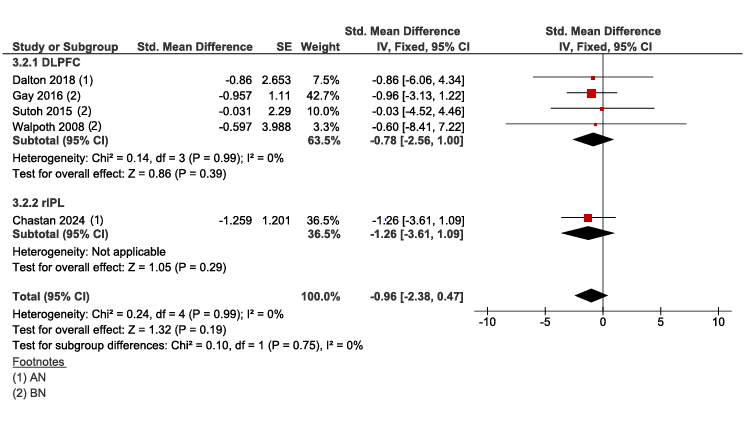


**Supplementary Figure 5.** Subgroup Analysis Based on Number of sessions for the Effect of rTMS on Depression – Single-Arm Analysis

*No statistically significant difference was observed between subgroups*


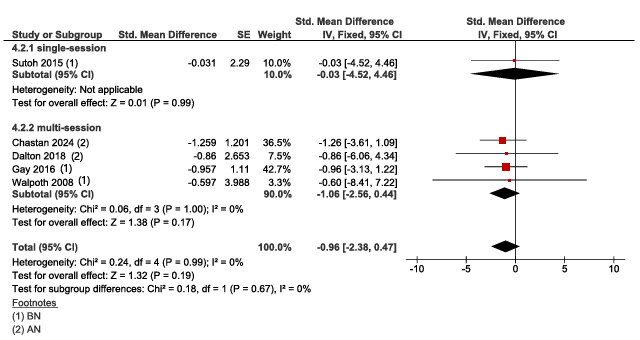


**Supplementary Figure 6.** Subgroup Analysis Based on Target Location for the Effect of rTMS on Anxiety – Single-Arm Analysis

*No statistically significant difference was observed between subgroups*


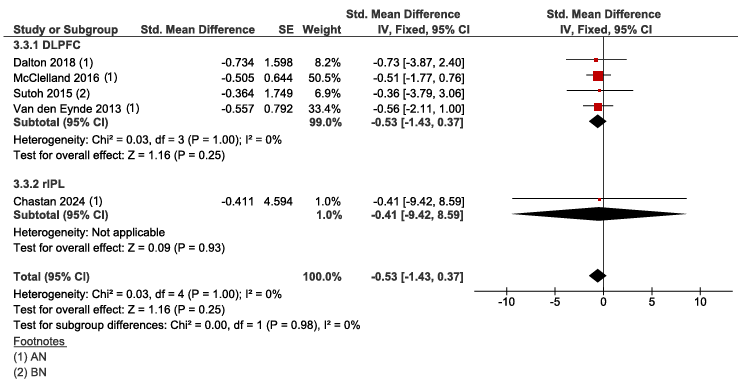


**Supplementary Figure 7.** Subgroup Analysis Based on Number of sessions on for the Effect of rTMS on Anxiety – Single-Arm Analysis

*No statistically significant difference was observed between subgroups*


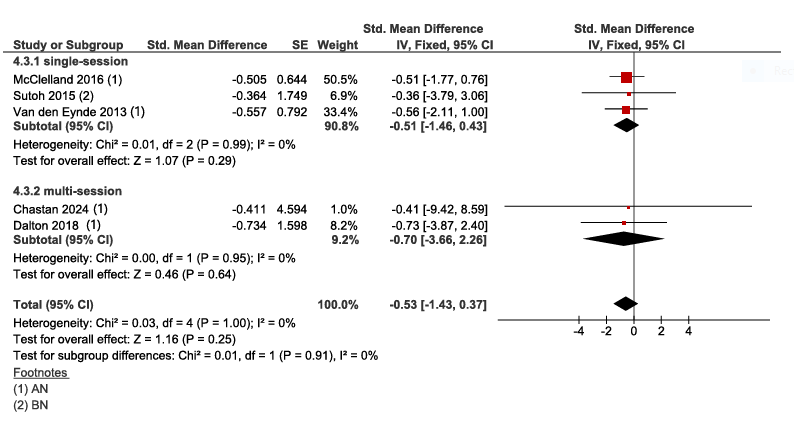


**Supplementary Figure 8.** Subgroup Analysis Based on Target Location for the Effect of rTMS on Binge Eating Frequency – Single-Arm Analysis (BN)

*No statistically significant difference was observed between subgroups*


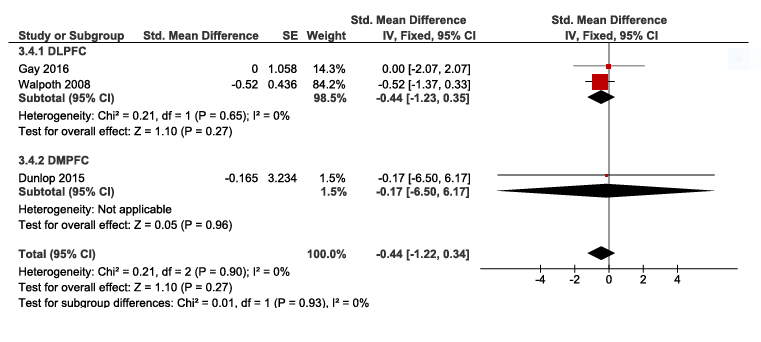

Supplement: Supplementary file 1 — Supplementary material 1. [file 12991_2026_673_MOESM1_ESM.docx]
